# Role of DHA in a Physicochemical Study of a Model Membrane of Grey Matter

**DOI:** 10.3390/membranes14120256

**Published:** 2024-12-03

**Authors:** Victor E. Cuenca, Viviana I. Pedroni, Marcela A. Morini

**Affiliations:** 1 Laboratory of Physical-Chemistry, Department of Chemistry, Universidad Nacional del Sur (UNS), Bahía Blanca 8000, Argentina; victor.cuenca@uns.edu.ar (V.E.C.); pedroni@criba.edu.ar (V.I.P.); 2 INQUISUR-CONICET, Bahía Blanca 8000, Argentina

**Keywords:** model membrane, grey matter, docosahexaenoic acid, lipid raft, molecular acoustic, zeta potential

## Abstract

The present study investigates a multicomponent lipid system that simulates the neuronal grey matter membrane, employing molecular acoustics as a precise, straightforward, and cost-effective methodology. Given the significance of omega-3 polyunsaturated fatty acids in the functionality of cellular membranes, this research examines the effects of reducing 1-palmitoyl-2-docosahexaenoylphosphatylcholine (PDPC) content on the compressibility and elasticity of the proposed membrane under physiological conditions. Our results align with bibliographic data obtained through other techniques, showing that as the proportion of PDPC increases in the grey matter membrane model, the system’s compressibility decreases, and the membrane’s elasticity increases, as evidenced by the reduction in the bulk modulus. These results could be interpreted in light of the emerging model of lipid rafts, in which esterified DHA infiltrates and remodels their architecture. We contend that the results obtained may serve as a bridge between biophysics and cellular biology.

## 1. Introduction

Docosahexaenoic acid (DHA) is the most common polyunsaturated fatty acid (PUFA) found in grey matter. Consuming DHA, whether through dietary sources or supplements, is essential due to its role as a vital nutrient linked to numerous health benefits, particularly in mental health. These benefits are thought to arise from DHA’s ability to modulate inflammatory processes in conditions such as cardiovascular diseases and Alzheimer’s disease [1,2,3,4,5]. Despite extensive research efforts, particularly involving two- or three-component model membranes, the molecular mechanisms underlying these effects are not yet fully understood. There is a growing consensus that lipid and protein distribution within membranes is non-random, featuring tightly packed regions known as lipid rafts composed of sphingolipids and cholesterol. These rafts play a crucial role in compartmentalizing signaling proteins that facilitate the function of membrane proteins [6,7,8]. The rafts hypothesis remains contentious, as there is limited direct quantitative experimental evidence supporting the existence of such domains, which are often referred to as putative domains [9,10]. Furthermore, one theory suggests that PUFAs are not incorporated into raft lipids due to their low affinity for cholesterol, which may prevent this incorporation and imply that they influence raft formation and function from outside these domains [11,12]. An emerging model [13] utilizing solid-state deuterium (²H) NMR spectroscopy to study a canonical raft mixture indicates that although PDPC exhibits a high degree of disorder, it can infiltrate ordered, raft-like domains and form small clusters within these regions. This fact supports the notion that n-3 PUFAs can influence protein activity by disrupting the clustering of rafts. Other studies have also identified DHA within rafts [14,15,16]. Nonetheless, the precise impact of n-3 PUFA on molecular organization within cellular rafts remains unclear. Some studies report increased membrane order [17], while others indicate a decrease [18] following treatment with n-3 PUFA. This discrepancy emphasizes the complexity of biological cells, where variations in the levels of other fatty acids can modulate the response.

In this study, molecular acoustics was employed to analyze the molecular organization of model multi-component membranes composed of six lipids. This configuration accurately reflects the lipid structure of neuronal membranes found in grey matter [19,20], maintaining realistic proportions. In this context, we examined the physicochemical effects of reduced PDPC content on neuronal membranes. We utilized ultrasound velocimetry and densitometry to determine sound speed and density within the membrane. This analysis provides insights into compressibility and phase states [21,22]. Complementary zeta potential measurements at varying temperatures provided insights into the phase state and surface charge of the lipid systems [23]. These techniques possess unique advantages over other methods, as they can assess changes across the entire lipid bilayer. They are highly accurate, non-intrusive, cost-effective, and require minimal sample volumes and concentrations. The morphology study of the proposed systems was conducted by Transmission Electron Microscopy and Dynamic Light Scattering. All experiments were done under physiological pH and temperature conditions, allowing for closer extrapolation to biological systems. This study elucidated the significance of omega-3 PUFA on a model membrane that simulates brain cell membranes. Furthermore, it provides evidence supporting the theory regarding the infiltration of these fatty acids into lipid rafts using straightforward methodologies.

## 2. Materials and Methods

### 2.1. Materials

**Figure 1:**
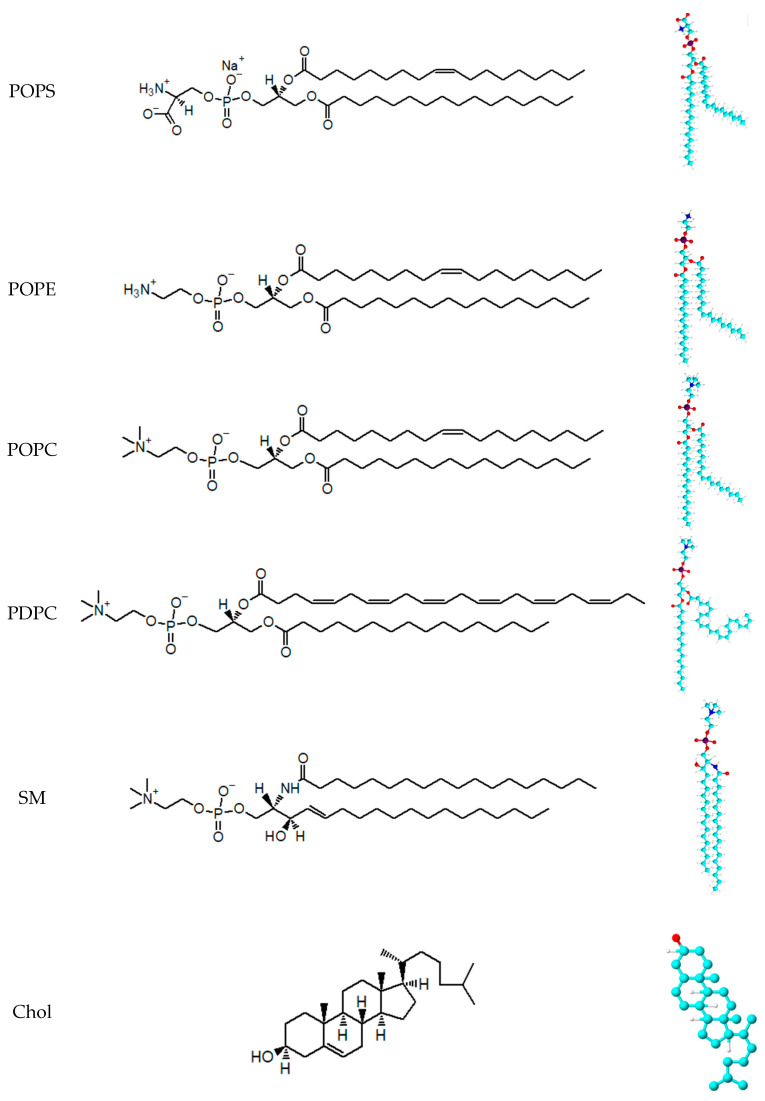
Structure and 3D representation of lipid used in this work.

1-hexadecanoyl-2-(9Z-octadecenoyl)-sn-glycero-3-phosphocholine (POPC), N-octadecanoyl-D-erythro-sphingosylphosphorylcholine (Brain SM), 1-hexadecanoyl-2-(9Z-octadecenoyl)-sn-glycero-3-phospho-L-serine (sodium salt) (POPS), 1-hexadecanoyl-2-(9Z-octadecenoyl)-sn-glycero-3-phosphoethanolamine (POPE),1-hexadecanoyl-2-(4Z,7Z,10Z,13Z,16Z,19Z-docosahexaenoyl)-sn-glycero-3-phosphocholine (PDPC), cholesterol (Chol) were acquired from Avanti Polar Lipids Inc. (Alabaster, AL, USA). Lipid structures and 3D representation are presented in Figure 1. All lipids were stored at −20 °C when not in use. 2-[4-(2-Hydroxyethyl)piperazin-1-yl]ethane-1-sulfonic acid (HEPES) and HEPES Sodium salt were purchased from Sigma-Aldrich (St. Louis, MO, USA). The Chloroform employed was of analytical grade. Finally, the mediums for lipidic dispersions were water (Ultrapure water, Super Q Millipore system, pH = 5.5, conductivity 5 mS.m^−1^) and HEPES Buffer solution (10^−2^ M, pH = 7.45 and conductivity 40 mS.m^−1^).

### 2.2. Model Lipid Membrane Preparation

Vesicles of POPC-Chol, POPC-POPE-Chol, POPC-POPE-SM-Chol, and POPC-POPE-SM-POPS-Chol were prepared by weighing appropriate quantities of each one to obtain proportions of interest. Vesicles of POPC-POPE-SM-POPS-PDPC-Chol were prepared in the same fashion, but PDPC quantities were measured using a Hamilton syringe from a 10 mg.mL^−1^ septum-sealed chloroform solution purged with nitrogen. All lipids were used as purchased without further purification. The molar ratios of lipids for six-component vesicles were calculated from the literature [19,20] to mimic a 55-year-old grey matter composition (ω-3_100_), for contrast systems were prepared to present omega deficiency with 50% (ω-3_50_) and 0% (ω-3_0_) of the original PDPC composition. Thus molar fraction composition resulted in POPC:POPE:SM:POPS:PDPC:Chol of 0.16:0.28:0.060:0.09:0.11:0.30 for ω-3_100_, POPC:POPE:SM:POPS:PDPC:Chol of 0.22:0.28:0.06:0.09:0.05:0.3 for ω-3_50_, and POPC-POPE-SM-POPS-Chol of 0.27:0.28:0.06:0.09:0.30 for ω-3_0_. For vesicles without PDPC compositions were as follows: POPC:Chol 0.7:0.30, POPC:POPE:Chol 0.35:0.35:0.30, POPC:POPE:SM:Chol 0.31:0.32:0.07:0.30, and POPC-POPE-SM-POPS-Chol 0.27:0.28:0.06:0.09:0.3. Molar fractions of lipids in the vesicles are calculated without considering the solvent.

To form the vesicular solutions, the masses of lipid corresponding to the proportion enunciated above were weighted separately with an error of ±1 × 10^−5^ g. All lipidic components were introduced in a test tube, then the lipids were dissolved in chloroform, and finally, the chloroform was removed by evaporation with a nitrogen stream to obtain a dry lipid film. The remaining traces of solvent were eliminated by a high vacuum system using a Thermo Scientific Speed Vac SPD11V (Thermo Fisher Scientific Inc, Waltham, MA, USA). The resulting dry lipid films were hydrated with 5 mL of Milli-Q water or 5 mL of HEPES buffer solution as required for the experiment. Sonication of samples was performed in an ultrasonic bath (70 W of power and 40 KHz of frequency) for 30 min to obtain large unilamellar vesicles (LUV). The dispersions acquired a final concentration of 2 mg.mL^−1^ for density, ultrasound velocity, and ZP experiments. Before conducting the density and ultrasound velocity measurements, the aqueous vesicle suspension was properly degassed by a vacuum system with constant agitation using a magnetic stirrer at 440 rpm.

Precautions were taken throughout the manipulation of PDPC-containing solutions to avoid oxidation. These precautions included limiting exposure to light, using a controlled atmosphere bag purged with high-purity nitrogen, and hermetic sealing the cuvettes during measurements. The degree of lipid oxidation was checked by FT-IR, and no oxidation was found. Remarkably, every sample was prepared at the moment of the measurements to avoid oxidation during storage.

### 2.3. Methods

#### 2.3.1. Ultrasound Velocity and Density

Molecular acoustic techniques allow access to solution properties in a non-invasive approach. Commercial density and sound velocity measurement instruments (Anton-Paar DSA 5000 densimeter and sound velocity analyzer, Graz, Austria) were used to obtain continuous, simultaneous, and automatic densities (*ρ*) and sound velocities (*u*). Measurements were taken at progressively decreasing temperatures. The speed of sound and density values are dependent on solution temperature; thus, this variable was controlled by the Peltier method within the equipment with a precision of ±0.01 °C. Two samples were prepared for each system. Density and sound velocity measurements were highly reproducible (superior to ±1 × 10^−5^ g cm^−3^ and ±0.01 m s^−1^, respectively).

The purpose of ultrasound velocity measurements is the evaluation of the elastic properties of the systems in the study. The adiabatic compressibility coefficient of solvent (*β*_0_) and aqueous solutions and suspensions (*β_S_*) was obtained using the following equation.

(1) $$\beta_{s}=1/u^{2}\rho$$

where *u* is the sound velocity of the suspension and *ρ* is the density [24], these parameters allow assessing the adiabatic compressibility coefficient of a liquid. The relative change in a physical characteristic per unit of solute concentration is more significant than its absolute value. Therefore, the concentration increments in sound velocity [*u*] must be calculated using the following formula:

(2) $$\left[u\right]=(u-u_{0})/(u_{0}c)$$

where *c* is the solute concentration (lipid) in mg/mL and *u* and *u*_0_ indicate the sound velocity solution and the solvent (distilled water or HEPES solution), respectively. Apparent specific partial volumes *φ_v_* were derived from the density data by:

(3) $$\varphi_{v}=[1-\frac{\left(\rho -\rho_{0}\right)}{c}](\frac{1}{\rho_{0}})$$

where *ρ* is the density of the solution, *ρ*_0_, is the density of the solvent, and *c* is the concentration of lipids in mg/mL. Variation of *β*_0_, [*u*], and *φ_v_* with temperature can be obtained by measuring changes in sound velocity and density. From a combination of specific volume and sound-velocity concentration increment measurements, the specific adiabatic compressibility, *φ_k_*/*β*_0_, of the liposomes was estimated:

(4) $$\frac{\varphi_{k}}{\beta_{0}}=-2\left[u\right]-\frac{1}{\rho_{0}}+2\varphi_{v}$$

The value of *φ_k_*/*β*_0_ represents changes in the volume compressibility of the vesicles relative to the solvent. Partial physical parameters describe the individual contributions of specific types of molecules to a measurable property. This is why these parameters can be used to link bulk properties to molecular features [25]. Thus, adiabatic compressibility of the lipid *β_lipid_* is given by

(5) $$\beta_{lipid}=\beta_{0}\left(2-\frac{\left[u\right]}{\varphi_{v}}\right)$$

the volumetric compressibility modulus *K_lipid_* is obtained by calculating the reciprocal of apparent adiabatic compressibility of the lipid:

(6) $$K_{lipid}=\frac{1}{\beta_{lipid}}$$

#### 2.3.2. Dynamic Light Scattering (DLS) and Zeta Potential

Non-invasive techniques were selected to study the size of the vesicles and superficial charge. DLS and Zeta potential techniques allow access to vesicle properties without sample disturbance. Zetasizer Nano ZS90 equipment (Malvern Instruments Ltd., Malvern, UK) was used to determine the sizes and Zeta Potential of liposomes. The measurements were performed at progressively decreasing temperatures, enabling the sample to attain thermal equilibrium and recording points every 2 °C with a stabilization period of 5 min (at ±0.1 °C constant temperature through the Peltier method). Two samples were prepared for each system. For size experiments, the hydrodynamic diameter is the result of ten independent measurements on each sample. For Zeta potential, twelve independent measurements were performed on each sample.

#### 2.3.3. Transmission Electron Microscopy (TEM)

To understand the morphology of the vesicles, a JEOL 100 CX II CCDGATANES 1000 W Erlangshen microscope (JEOL Ltd., Akishima, Japan) was used with 50,000 magnifications over a carbon-coated copper microscopy grid (400 mesh). An aqueous uranyl acetate solution was used to stain the samples; then, they were washed with water and placed in a Petri dish to dry. This method has the disadvantage of polluting the sample with undissolved uranyl acetate crystals. However, these objects are easily distinguishable for their angular crystalline structure and high absorption; thus, they do not mask the results. Analysis of the images was performed following published protocols [26].

#### 2.3.4. Statistical Analysis

A two-tailed, unequal variance *t*-test was used to compare the roundness and the size of the vesicles in TEM conditions and the hydrodynamic diameter obtained by DLS. T values were calculated using the following formula:

(7) $$T\text{-}value=\frac{mean1-mean2}{\sqrt{\left(\frac{var1}{N1}+\frac{var1}{N2}\right)}}$$

where *mean*1 and *mean*2 are the average values each of the sample set, *var*1 and *var*2 are the variance of the sample set, and *N*1 and *N*2 are the number of records in each sample set. The following formula was used to calculate the degree of freedom (DF):

(8) $$DF=\frac{{\left(\frac{var1}{N1}+\frac{var2}{N2}\right)}^{2}}{\frac{{var1}^{2}}{{N1}^{2}(N1-1)}+\frac{{var2}^{2}}{{N2}^{2}(N2-1)}}$$

To evaluate the statistical significance of the results, the *p*-value was used with a significance level α set at 0.05.

All the results in this work are presented with the confidence interval: $mean\pm \frac{c\cdot S}{\sqrt{N}}$, where *c* is the critical value, 1.96, *S* is the standard deviation, and *N* is the number of records in the sample set.

## 3. Results and Discussion

After an extensive search on the subject, the most detailed works, not only on the lipid proportion of the normal human brain but also on its fatty acid composition, are those of O’Brien and Sampson [19,20]. Of the four age groups that present these works, we focus on the adult brain, obtaining the proportion of lipids esterified with DHA. Although other polyunsaturated fatty acids are present, the aforementioned omega-3 is the most abundant. As a result of our reference choice, a multicomponent lipid model membrane was designed to mimic the neuronal membrane of grey matter. The physicochemical study of these systems will mainly be approached from the mechanical perspective by molecular acoustics and complemented with the electrical perspective by zeta potential.

Although the model membranes are simplified, they provide insights into biological membranes and cellular processes. The biological importance of the experimental techniques applied in this work is briefly discussed below.

Importance of Studying Molecular Acoustics in Biological Membranes

Membranes contain over one thousand different lipid species, yet, besides a few exceptions, the clear function of each species remains greatly unknown. Among those, phospholipids constitute the major family, and while most cellular membranes contain saturated and monounsaturated acyl chains, certain organs like the brain possess about 30% of polyunsaturated fatty acid phospholipids (PUFA-PLs) with synaptic vesicles containing up to 80% of phospholipids with at least one polyunsaturated acyl chain [19,20]. The potential of ultrasonic velocimetry in biomolecular studies has become clear only in recent years because of the development of new high-precision measurement methods. This technique is used with the aim of investigating mechanical and structural properties, such as the compressibility and elasticity of cell membranes and their components. The membrane activity modulating role of lipids could be unveiled by the overall compressibility of the system [27]. For example, membrane thickness, related to elasticity and compressibility, is critical for facilitating membrane proteins’ proper insertion, function, sorting, and inheritance [28].

Importance of Studying Zeta Potential in Cellular Membranes

It has been shown that the membrane potential (Vm) mechanically and dynamically alters the zeta potential (ζ), the electrical potential measured by a few nanometers from the cell surface, or the extracellular potential. This potential defines how the cell interacts with charged entities in its environment, including ions, molecules, and other cells, which could help explain electrophysiological behaviors related to cellular function regulation. The aforementioned suggests that changes in electrical properties are associated with changes in cellular behavior, offering potential new insights in membrane biophysics, cellular electrophysiology, cell function, and the growing field of bioelectricity [29].

### 3.1. Consecutive Addition of Lipids

**Figure 2:**
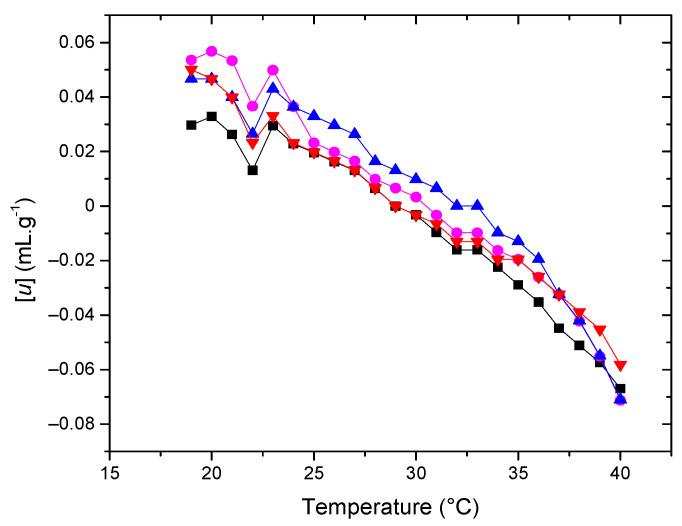
Relative concentration increment of sound velocity [u] (■) POPC + Chol (●) POPC + POPE + Chol (▲) POPC + POPE + SM + Chol (▼) POPC + POPE + SM + POPS + Chol in water. Error bars not shown to avoid cluttering.

**Figure 3:**
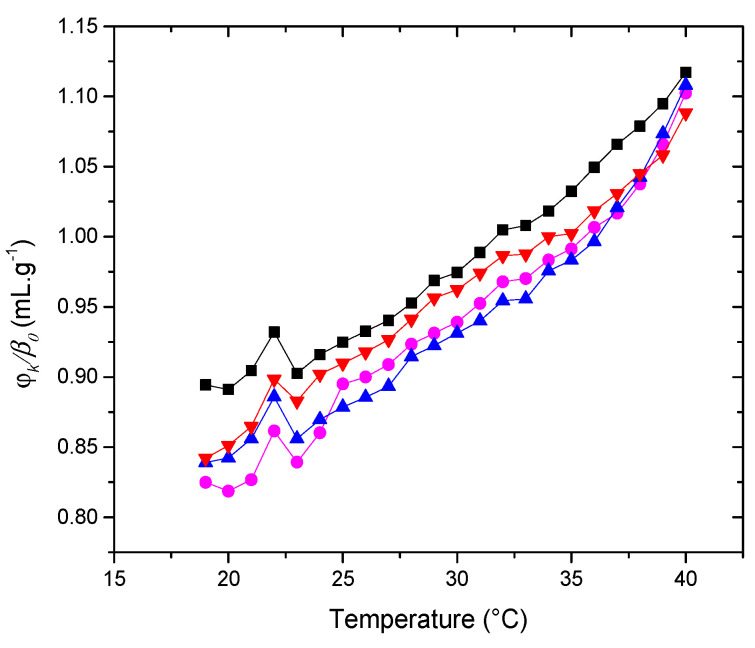
Adiabatic compressibility, *φ_k_*/*β*_0_, of the liposomes (■) POPC + Chol (●) POPC + POPE + Chol (▲) POPC + POPE + SM + Chol (▼) POPC + POPE + SM + POPS + Chol in water. Error bars not shown to avoid cluttering.

Given that its number of lipid components results in a complex model membrane, we conducted a systematic study starting with a bicomponent model membrane and adding one lipid at a time to observe the contributions of each to the membrane’s mechanical properties. The following systems, ranging from two to five components, were studied: POPC-Chol; POPC-POPE-Chol; POPC-POPE-SM-Chol; and POPC-POPE-SM-POPS-Chol. The respective proportions are depicted in the experimental section. The different samples’ specific volume and adiabatic compressibility were calculated from density and sound velocity measurements. The results for the increment in ultrasound velocity concentration, [*u*], and the specific adiabatic compressibility coefficient, *φ_K_*/*β*_0_, for the different systems are presented in Figure 2 and Figure 3 as a function of temperature.

The sound velocity value shows a characteristic dip at the transition temperature [30,31,32] An increase in [*u*] is typical for the temperature region above Tm. This trend might be due to a decrement in lipid bilayer ordering, presumably related to increased conformational freedom of the phospholipid hydrocarbon chains as the temperature rises [33]. This characteristic shape of the [u] vs. T plot, along with the nature of the minimum in the [*u*] value, has been documented in various works focusing on ultrasound velocimetry studies of temperature-induced phase transitions in vesicle suspensions composed of saturated PCs [21,30,34,35]. As shown in Figure 2, the typical dip in the [*u*] vs. temperature plot is not as pronounced as observed in pure lipids due to the presence of cholesterol in all systems, making the transition less cooperative. Still, this dip can be observed between 22 and 23 °C.

Since changes in [*u*] include changes in the compressibility of both the bilayer and the hydration shell, further analysis of the membrane’s mechanical properties requires evaluating changes in the specific volume of the liposomes. The density of the liposome suspensions can be determined for this purpose [33]. With such density values and employing Equation (3), the specific volume, *φ_V_*, was calculated. Based on the [u] and *φ_V_* values obtained, we calculated the specific adiabatic compressibility, *φ_K_*/*β*_0_, (Equation (4)) for the studied systems. These results are shown in Figure 3. An increase in specific compressibility with temperature is evident in all the systems and reflects the enhanced disorder in the liposomes (mainly due to the behavior of the hydrophobic phase of the bilayer) and an increment in the compressibility of the hydration shell as temperature rises [21]. The degree of order reflects the anisotropy in molecular motion.

The curves showing the dependence of *φ_k_*/*β*_0_ exhibit similar shapes. Regarding the magnitudes, there is a significant difference between the system containing only POPC + Chol and the rest of the systems (at 37°, *p* < 0.03774), whereas the magnitudes are similar in the membranes containing POPE (at 37°, *p* > 0.2055). This observation indicates that this group of lipids, which share the same hydrophobic composition and head groups, does not create a structural difference that significantly alters the compressibility of the vesicles as lipid species are added. This is particularly noteworthy since all samples contain the same ratio of cholesterol. The next section will revisit this conclusion when examining the effect of adding PDPC on the compressibility of the system.

### 3.2. Membranes with PDPC

In this section, we will discuss the results obtained from molecular acoustics, zeta potential measurements, dynamic light scattering (DLS), and transmission electron microscopy (TEM) of the POPC-PDPC-POPE-SM-POPS-Chol systems, with varying concentrations of PDPC (0%, 50%, and 100%). The main objective is to analyze how these changes affect the membrane’s mechanical properties, surface charge characteristics, and vesicle morphology. These variations were evaluated in a HEPES buffer medium and across a temperature range that includes the physiological temperature of 37 °C. As noted in Section 3.1, the experimental data presented below pertain to the membrane in a fluid phase.

#### 3.2.1. Molecular Acoustics

**Figure 4:**
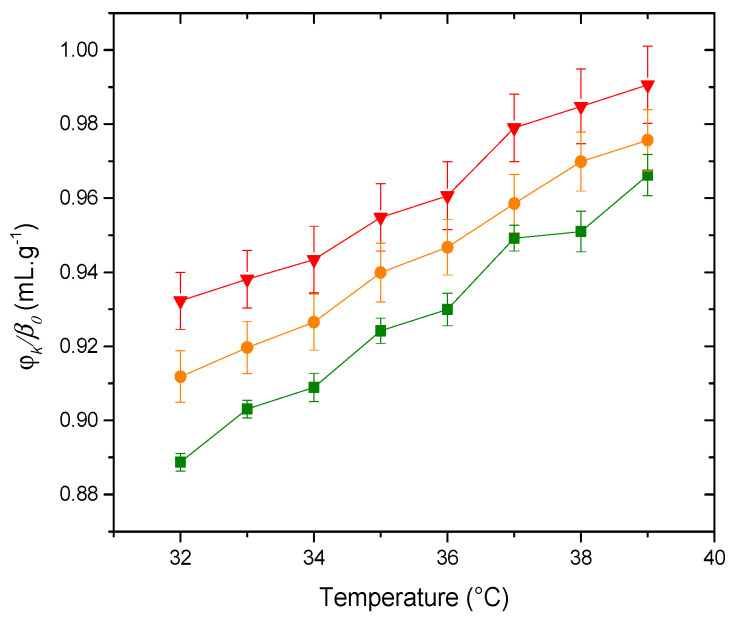
Adiabatic compressibility, *φ_k_*/*β*_0_, of the liposomes (▲) PDPC 0% (●) PDPC 50% (■) PDPC 100% in HEPES solution.

Figure 4 shows curves of *φ_K_*/*β*_0_ vs. temperature for three types of vesicles. The membrane composed of 100% PDPC is the least compressible, rendering it the most compact. Initially, one might assume that altering the proportion of PDPC in the membrane would not significantly impact its compressibility. However, it is crucial to recognize that these membranes are aqueous dispersions, similar to those found in cells, which are inherently low-compressibility systems. In such contexts, even minor variations in compressibility values can be physically significant [36].

As shown in Figure 4, the liposomal dispersion containing 0% PDPC exhibits the highest compressibility. The decrease -with PDPC incorporation- in specific compressibility (at 37 °C *p* (0–100%) = 0.02428) reflects the corresponding enhanced order in the membranes. This result is surprising given the particular molecular structure of the ω-3 polyunsaturated fatty acid. However, a previous study conducted by our research group with the DPPC-DHA system showed similar behavior. In that study, the system was analyzed using, among other techniques, computer simulation, which provided the membrane’s order parameter (SCD). The DPPC membrane with the presence of DHA shows higher SCD values than the pure membrane [37]. This order increase, as compared to pure DPPC, follows a similar qualitative trend as that found for the case of esterified DPPC-DHA membranes [38]. Although these systems are binary and simpler than those used in the present work, similar trends have been found in systems containing sphingomyelin and cholesterol, like ours [13]. In this case, the behavior of PDPC can be understood through its incorporation into lipid rafts—regions densely packed with sphingolipids and cholesterol. The stability of these rafts originates from the structural compatibility between the mostly linear arrangement of saturated sphingolipid chains and the flat, tetracyclic structure of cholesterol. Additionally, hydrogen bonding between the hydroxyl group on cholesterol and the amide group in the sphingosine backbone of sphingolipids further reinforces their stability. In contrast, polyunsaturated phospholipids have physical properties that, in many ways, are the opposite of those of sphingolipids [8]. The low energy barrier to rotation around the single bonds in the =CH-CH_2_-CH= repeating unit in a PUFA chain allows rapid isomerization through various conformations [39]. These structural fluctuations push the rigid steroid moiety of cholesterol away, and to prevent close proximity, polyunsaturated phospholipids segregate into highly disordered domains that are depleted of cholesterol, which are the complete opposite of lipid rafts. Kinnun et al. [13] concluded that, despite the high disorder and aversion to cholesterol, a significant amount of polyunsaturated phospholipid infiltrated SM-rich/cholesterol-rich raft-like domains. In that study, most of the lipid, relative to the total lipid, was found in the raft-like domain. Despite the substantial infiltration of PDPC into the raft-like domains, there appears to be only a minimal effect on the ordering of the SM, suggesting the presence of an internal structure that limits the contact between SM and PDPC. In summary, our results align with the bibliographic data found, meaning that as the proportion of PDPC increases in the grey matter model membrane, more of it is incorporated into the SM-Chol raft, resulting in a more compact structure that decreases the compressibility of the system.

In the context of nanomechanics, elasticity is a notable physical property that has garnered recent interest due to its role at the nano-bio interface. In this work, ultrasound velocimetry and densitometry were used to assess volumetric elasticity modulus or bulk modulus as a tool to characterize membrane elasticity [40]. One of the main advantages of the presented technique is that the surface remains unaltered throughout the entire measurement process, that is, without the artifacts by adsorption onto a substrate and the effect of a probe. Another strength highlighted in our proposal is that the obtained elasticity modulus does not depend on a physical model, such as the Hertz model for atomic force microscopy (Young’s modulus), and the required considerations, such as assuming the sample is homogeneous and isotropic. Based on the above, the bulk modulus obtained through ultrasonic velocimetry provides a genuine measure of the membrane elasticity of the system under study. Additionally, this technique requires a sample of small volume and low concentration.

**Figure 5:**
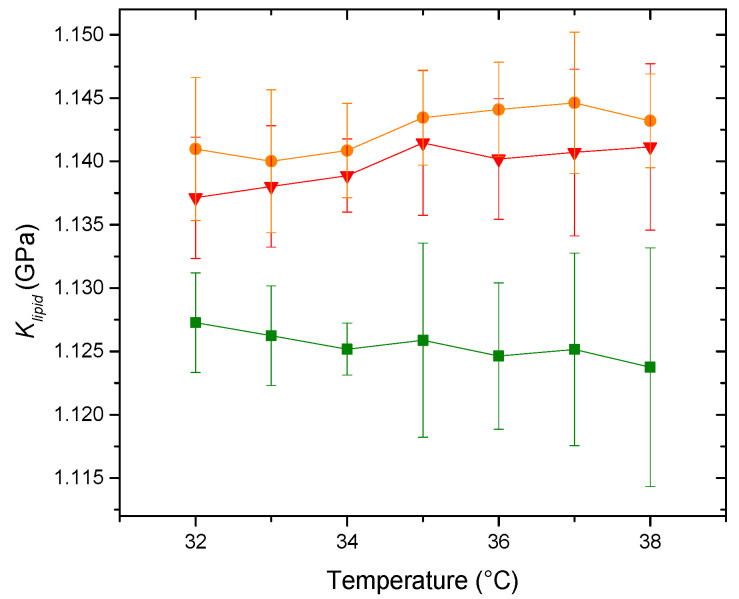
Elastic modulus, *K_lipid_*, of the liposomes (▲) PDPC 0% (●) PDPC 50% (■) PDPC 100% in HEPES solution.

In complex systems where no single region is representative of the whole, it is crucial to assess mechanical properties comprehensively, considering the entirety of the nanoparticle. Given the membrane’s mechanical properties and anisotropy, a thorough understanding of these properties necessitates investigating membrane deformation in various directions [41]. Figure 5 shows the behavior of the elastic modulus as a function of temperature for the three systems with varying PDPC content.

**Figure 6:**
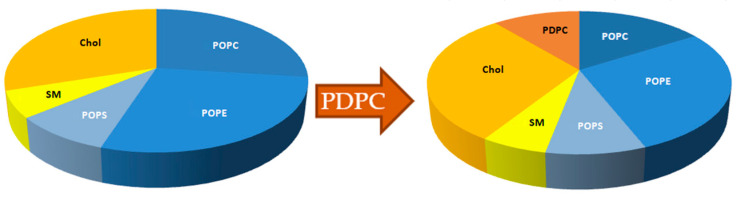
Qualitative diagram of the proposed results obtained through molecular acoustics.

Our molecular acoustics experiments initially reveal a subtle but significant increase in the elasticity of the membrane containing the maximum PDPC when compared with the system with 50% PDPC (at 37 °C *p* = 0.04506), as shown by the decrease in the bulk modulus. This behavior aligns with previous studies that highlight increased bending flexibility or elasticity in giant unilamellar vesicles containing PUFAs [42,43,44,45]. The incorporation of PUFA-PLs into membranes alters their elasticity, resulting in a decrease in thickness by approximately 0.1 nm [42,43]. While this work does not include a specific measurement related to membrane thickness, we can infer from the elasticity measurements that incorporating PDPC into the membrane would have the same effect on thickness. Based on the proposals by Kinnun et al. [13] regarding this matter, there are two possible scenarios for the arrangement of PDPC into an SM-rich/chol-rich ordered domain. PDPC molecules cluster in small subdomains within a larger raft-like domain in one scenario. In another scenario, PDPC molecules accumulate at the edge of this raft-like domain, resulting in a gradient of concentration and thickness at the boundary with the thinner PDPC-rich and cholesterol-poor regions. The proposed model by Kinnun et al. [13] allows us to correlate the data obtained from compressibility and elasticity in grey matter neuronal membranes while supporting the theory that PDPC incorporates into SM-Chol domains, as shown in the qualitative diagram of Figure 6.

Many measurement techniques reported in the literature [46] for studying membrane order and elasticity include various methods, such as the fluorescence of lipophilic dyes, which respond to membrane order, and atomic force spectroscopy, which quantifies the force required to indent the lipid bilayer. Additionally, deuterium (^2^H) solid-state NMR spectroscopy is used to assess the molecular order of lipid acyl chain segments. Fluorescence correlation spectroscopy evaluates the diffusivity of probes within the membrane, while Raman spectroscopy examines how spectral intensity ratios are influenced by acyl chain order. Gupta et al. [46] point out that their findings expose inconsistencies across various measurement techniques, which at times produce contradictory results. Accordingly, they propose that no single probe is sufficient to reliably determine membrane order. This underscores the significance of our proposal to employ molecular acoustics as a means of evaluating both the order and elasticity of a given membrane system.

#### 3.2.2. Zeta Potential

**Figure 7:**
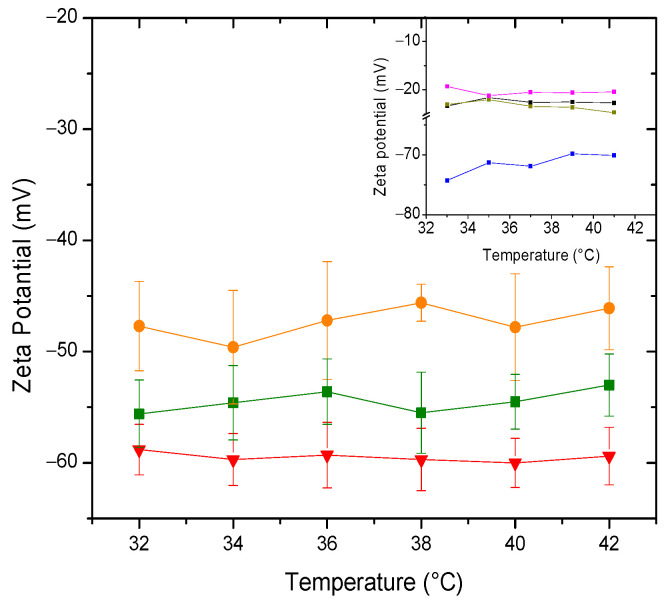
Zeta potential of (▲) PDPC 0% (●) PDPC 50% (■) PDPC 100% in HEPES solution. Insert: Zeta Potential of (■) POPC + Chol (■) POPC + POPE + Chol (■) POPC + POPE + SM + Chol (■) POPC + POPE + SM + POPS + Chol in water.

This section presents the results and analyzes the technique used, both from the perspective of surface charge originating from the surface arrangement of the lipid head groups. Zeta potential provides information about the liposomal surface through surface charge. The sign of the zeta potential for a zwitterionic lipid in an aqueous medium, as a consequence of the surface arrangement of head groups (and not due to surface adsorption of ions from the medium), was proposed by our research group [47]. It is considered that the sign of the zeta potential of the different proposed systems is a consequence of surface rearrangements, including domains, resulting from the different interactions and affinities between the components of the system. In the inset of Figure 7, it can be observed that starting with the bicomponent system, POPC-Chol, it shows negative zeta potential values. Based on the planar structure of cholesterol, which allows intercalation in the acyl chain region of phospholipids, Pandit et al. [48] proposed the formation of complexes between DPPC and cholesterol, where not only is the typical OH-O hydrogen bond established (cholesterol is a donor and phospholipid is an acceptor), but also between the methyl hydrogens of the phospholipid’s choline group and the oxygen atom of the hydroxyl group of cholesterol (cholesterol as an acceptor and phospholipid as a donor). The mentioned interactions may result in the exposure of the phosphate groups on the surface, leading to a negative zeta potential for the POPC-Chol system. With the incorporation of POPE, compared to the previous system, the unsaturation of the hydrocarbon tails is maintained, while the PE head group does not alter the surface charge, as it is also a zwitterionic lipid. This results in electrical behavior similar to that of the binary system. At the surface level, the addition of SM does not introduce significant modifications to the behavior of the new system. Although the hydrocarbon tails are more rigid, the head group remains zwitterionic. In summary, with the successive addition of other lipids, the result in terms of the sign and magnitude of the zeta potential is similar. It is clearly observed that the addition of POPS drastically makes the membrane potential more negative, as expected for a charged lipid. With this last system as a point of reference, the behavior of surface charge with the addition of PDPC in buffer solution is analyzed and illustrated in Figure 7.

The PDPC 0% membrane prepared in HEPES buffer shows zeta potential values clearly less negative than those in water (see insert in Figure 7). These changes are a consequence of the ionic medium´s effect. It is worth noting that DHA is esterified and, therefore, unable to ionize and contribute charge to the surface. This fact is relevant because, when DHA is free, its behavior differently affects the corresponding membrane [37]. The magnitude of the potential of the system with 50% PDPC differs slightly from the rest of the systems (at 37 °C, *p* (0–50%) = 0.001070 and *p* (50–100%) = 0.03852), although it is evident that the addition of PDPC alters the structuring of the vesicular surface.

This rearrangement may result from the effect of polyunsaturated fatty acids on SM-Chol domains. Considering the previous discussion regarding the decrease in adiabatic compressibility in these systems upon the addition of PDPC and the membrane model proposed by Kinnun et al. [13], the reorganization of the raft after the inclusion of the fatty acid leads to greater surface exposure of the rigid sphingomyelin compared to mono- or polyunsaturated lipids. This effect results in a less negative zeta potential in the systems containing PDPC.

#### 3.2.3. Morphological Study

##### DLS

**Figure 8:**
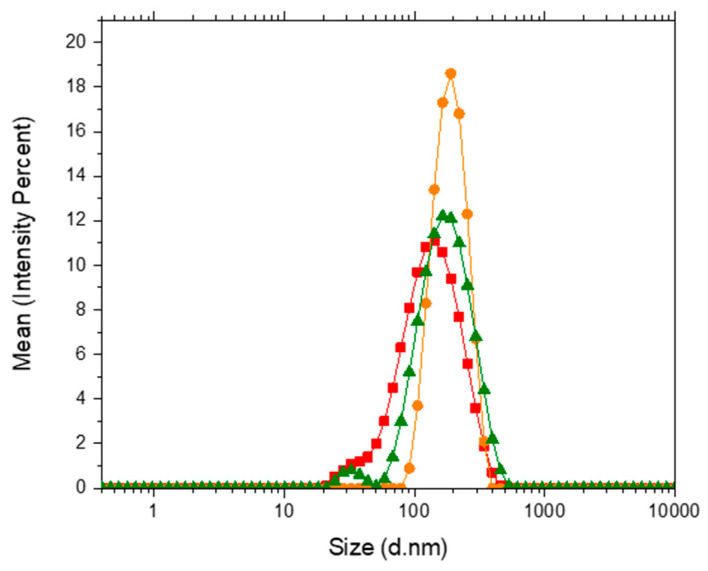
Apparent hydrodynamic diameter distribution for (■) 0% PDPC (●) 50% PDPC (▲) 100% PDPC.

The intensity-weighted size distribution was used to characterize the size of the vesicles formed in water and in the HEPES buffer solution. Vesicles containing POPC-POPE-SM-POPS-Chol in water presented an apparent hydrodynamic diameter of 455 ± 70 nm. The system is stable in the temperature range studied, and the results are highly reproducible. In the HEPES buffer solution, the apparent hydrodynamic diameters for the systems containing 0%, 50%, and 100% PDPC were 145.8 ± 35.9 nm, 192.9 ± 28.9 nm, and 187.5 ± 43.7 nm, respectively. Size distributions are presented in Figure 8.

Results indicate that vesicles formed in HEPES buffer solution present no significantly different apparent hydrodynamic diameters (*p* (0%–50%) = 0.05264, *p* (50%–100%) = 0.8406 and *p* (0%–100%) = 0.8401). Conversely, vesicles formed in water show a much larger apparent hydrodynamic diameter. This result indicates that the HEPES buffer allows the formation of smaller vesicles, most probably because of the compensation of charges at the internal and external surfaces of the liposomes. DLS technique is sensitive to size distribution as intensity analysis is biased towards larger particles, for the scattering intensity is proportional to the radius to the sixth power (Rayleigh scattering). Furthermore, the correlation function analysis results in the translational diffusion coefficient of the equivalent sphere, not allowing access to vesicular morphology.

##### TEM

**Figure 9:**
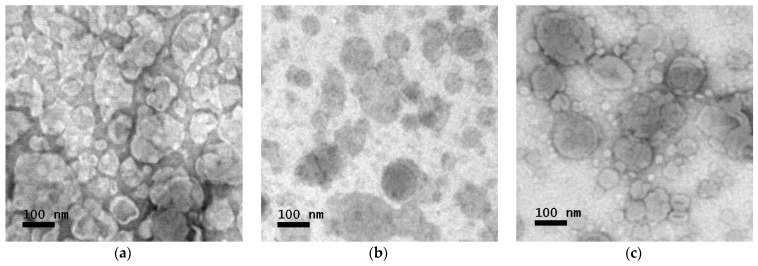
TEM image of vesicles with (**a**) 0% PDPC, (**b**) 50% PDPC (**c**) 100% PDPC.

**Figure 10:**
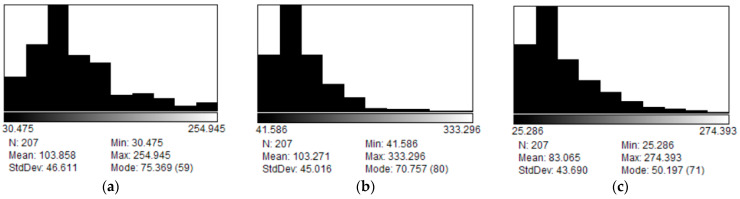
Diameter distribution of vesicles with (**a**) 0% PDPC, (**b**) 50% PDPC (**c**) 100% PDPC.

The TEM technique was utilized to study the system and gain insights into the shape of the vesicles. Images of the vesicles were taken to observe the morphology and to obtain the liposome size distribution (Figure 9). The sizes of the vesicles were found to be 103.8 ± 6.3 nm for the system with 0% PDPC, 103.3 ± 6.1 nm for the system with 50%PDPC and 83.1 ± 6.0 nm for the system with 100% PDPC (Figure 10). The system containing 100% PDPC presents significant differences in diameter with those with 0% (*p* = 3.903 × 10^−6^) and 50% PDPC (*p* = 4.912 × 10^−6^). No significant difference in diameter is found between the systems with 0% and 50% (*p* = 0.9323). It is well-known that cholesterol increases the rigidity of fluid membranes through increased molecular ordering, and there is an important linkage between unsaturation and an increase in membrane fluidity [49]. It has been demonstrated that the size of LUVs increases with membrane rigidity [50]; thus, the systems with low amounts of PDPC are expected to present the largest diameters.

Sizes obtained with TEM are smaller than those obtained with DLS. However, this result is predictable, considering DLS provides the apparent hydrodynamic diameter of the vesicles and not the actual diameter, and contribution in scattering from larger particles shifts the resulting size distribution towards larger diameters. Another critical factor is that samples inside the TEM are exposed to high vacuum and heating from the electron beam. In these conditions, water bound to the vesicles would be removed, reducing the liposomal size and producing the collapse of the vesicles.

Vesicles are expected to be spherical in shape with some irregularities due to collapse from contact with the grid and dehydration from the high vacuum in the microscope chamber. However, only samples containing PDPC presented this characteristic. To study this change in morphology, the roundness of the vesicles in TEM images were analyzed using the roundness formula.

(9) $$Roundness=\frac{4*area}{\pi *{\left(major\;axis\right)}^{2}}$$

Roundness was found to be 0.698 ± 0.019, 0.838±0.013, and 0.832 ± 0.012 for the system with 0% PDPC, 50%PDPC, and 100% PDPC, respectively (*n* > 200 for each sample,). Significant differences in roundness were found between the sample with no PDPC and those with PDPC (*p* = 1.020 × 10^−26^ for 0–50% and *p* = 7.611 × 10^−26^ for 0–100%), while no difference was found between the systems with PDPC (*p* = 0.5232 for 50–100%). Literature indicates that polyunsaturated lipids increase membrane fluidity [51], and the difference in the roundness for the system with 0% PDPC could be explained, considering that PDPC increases this parameter. Biological membranes consist of patches of differing composition called domains. Lipid domains are a consequence of preferred affinities between different lipids. The membrane´s fluidity is tied to the presence of omega-3 PUFA and might raise differences in liposome morphology in TEM conditions. The presence of PDPC would induce the formation of larger cholesterol domains that remain spherical during collapse [52]. On the other hand, in the absence of PDPC, small regions of high rigidity would form and give rise to a more angular shape in TEM conditions. Membrane fluidity is crucial in proteins’ activity in the bilayer [53], and PDPC would have an influence on increasing domain fluidity. The data obtained from the analysis of the morphology of the proposed vesicles align both with the results obtained through molecular acoustics regarding adiabatic compressibility and with bibliographic information [51,52,53].

Since our work aims to serve as a bridge between biophysics and cellular biology, we present, as a corollary, a table summarizing the characteristics of the membranes studied under physiological conditions (Table 1).

## 4. Conclusions

The present work contributes to understanding how one polyunsaturated omega-3 fatty acid, crucial in diverse cellular processes and pathologies, impacts the mechanical properties of a model membrane, studied by molecular acoustics, techniques not previously used for this purpose. We analyzed a multicomponent lipid system that mimics a grey matter neuronal membrane. These simple, cost-effective, and precise experimental techniques allowed us to study the system. Our results show more favorable membrane characteristics with a higher proportion of PDPC for forming the ordered domains, which are biologically essential for the insertion of membrane proteins. Our work’s conclusions align with the proposal of the emerging model, which has ω-3 PUFAs remodeling the architecture of lipid rafts, enriched in sphingolipids and cholesterol. Future work will examine the effect of dietary supplementation of this polyunsaturated fatty acid and other omega-3 PUFAs.

## Figures and Tables

**Table 1:** Summary of the physicochemical properties analyzed for model grey matter membranes under physiological conditions.

Physicochemical Properties	0% PDPC	50% PDPC	100% PDPC
Adiabatic Compressibility (g.mL^−1^)	0.979 ± 0.009	0.959 ± 0.008	0.949 ± 0.004
Elastic Modulus (GPa)	1.140 ± 0.006	1.144 ± 0.005	1.125 ± 0.008
Zeta Potential (mV)	−59.5 ± 4.1	−46.4 ± 5.5	−54.5 ± 4.7
Hydrodynamic Diameter (nm)	145.8 ± 35.9	192.9 ± 28.9	187.5 ± 43.7
Vesicular Diameter (TEM, nm)	103.8 ± 6.3	103.3 ± 6.1	83.1 ± 6.0

## Data Availability

The authors can provide the employed data on demand.
